# Progress of Wearable and Flexible Electrochemical Biosensors With the Aid of Conductive Nanomaterials

**DOI:** 10.3389/fbioe.2021.761020

**Published:** 2021-11-22

**Authors:** Tahir Raza, Lijun Qu, Waquar Ahmed Khokhar, Boakye Andrews, Afzal Ali, Mingwei Tian

**Affiliations:** ^1^ Research Center for Intelligent and Wearable Technology, College of Textiles and Clothing, State Key Laboratory of Bio-Fibers and Eco-Textiles, Intelligent Wearable Engineering Research Center of Qingdao, Qingdao University, Qingdao, China; ^2^ College of Physics, Qingdao University, Qingdao, China; ^3^ Ocean University, Qingdao, China

**Keywords:** electrochemical biosensor, conductive nanomaterials, non-invasive detection, flexibility, immunosensors, DNA biosensors

## Abstract

Conductive nanomaterials have recently gained a lot of interest due to their excellent physical, chemical, and electrical properties, as well as their numerous nanoscale morphologies, which enable them to be fabricated into a wide range of modern chemical and biological sensors. This study focuses mainly on current applications based on conductive nanostructured materials. They are the key elements in preparing wearable electrochemical Biosensors, including electrochemical immunosensors and DNA biosensors. Conductive nanomaterials such as carbon (Carbon Nanotubes, Graphene), metals and conductive polymers, which provide a large effective surface area, fast electron transfer rate and high electrical conductivity, are summarized in detail. Conductive polymer nanocomposites in combination with carbon and metal nanoparticles have also been addressed to increase sensor performance. In conclusion, a section on current challenges and opportunities in this growing field is forecasted at the end.

## Introduction

Today we live in the new era of the internet of things (IoT), where everything is connected, and smart objects like sensors and actuators can communicate with each others as well as generate and exchange information (Li et al., 2017). Wearable sensors are attached to the human body to assist in the constant measurement of the wearer’s health and fitness to help physicians and nurses observe patients, prevent illness, and monitor the health of babies at home (Byrne et al., 2010; Tao et al., 2011; Liu et al., 2012; Brodt et al., 2013; Wen and Eychmüller, 2016; Jang and Han, 2017; Qu et al., 2018). Their potential role in handling the emerging pandemic The COVID-19 outbreak has recently gotten much attention (Adans-Dester et al., 2020). Various studies relate Covid-19 to physiological parameters such as comforting heartbeat, breathing rate, skin temperature, blood oxygen saturation, and compatibility. wearable sensors are also being supported and could assist in identifying an event-related viral infection/spread early, and therefore, allowing timely interference to stop imminent spread (Seshadri et al., 2020). Wearable sensors and fitness controllers that detect vital signs and physical moments such as heart rate and blood pressure are also commercially available (Fitbit, WHOOP strap or Apple Watch). In addition to textile-based sensors (Farajikhah et al., 2020), transdermal alcohol vapour sensors with patch-like sensors were also introduced in 1992 (Swift et al., 1992). Although wearable sensors for physiological data collection have been widely used in medical and consumer goods. Even though there was a 0.5–2 h delay in detection by using body vapour, which encouraged researchers to switch to other methods of detection such as sweat (Schazmann et al., 2010), saliva, tear (Chu et al., 2011) and interstitial fluid (Rebrin et al., 2010; Schazmann et al., 2010). Wearable chemical (electrochemical and biochemical) sensors can offer extensive molecular data in various sectors, including medical, sports, nutrition, fitness, and defence (Fraser et al., 2011; Yang and Gao, 2019). The development of wearable electrochemical biosensors has accelerated in recent decades (Bandodkar et al., 2015b; Kim et al., 2017). These kinds of wearable electrochemical biosensors can non-invasively measure the dynamic variation of biochemical markers in biological fluids such as sweat, saliva, and interstitial fluid (Alizadeh et al., 2018; Kim et al., 2019). Although the latest developments in wearable electrochemical biosensors are achieved through the significant contributions of many influential researchers worldwide. Among the numerous sensing techniques, electrochemical source offers several benefits, including its simple structure with high sensitivity, fast reaction, and low power consumption (Mamalis et al., 2004; Meyyappan, 2004; Pérez López, 2009; Mpanza, 2016). It is widely acknowledged that conventional sensing electrodes are the basic components and play a dominant role in wearable electrochemical biosensors (Lee et al., 2016; Zhu et al., 2019). Recently, nano-structural materials, such as metal nanoparticles, carbon nanomaterials, and conductive polymers, have drawn much interest due to their unique electrical, physical, and chemical properties, as well as their high biocompatibility. Which are being used as sensing electrode materials in wearable electrochemical biosensors as compared to bulk materials (Imani et al., 2016; Lee et al., 2016; Bandodkar et al., 2017; Jeerapan et al., 2020; Li and Wen, 2020). The inherent properties of multidimensional nanomaterials, such as stretchability, provide excellent stability to sensors, which is essential for wearable applications (Feng and Zhu, 2019). Furthermore, the porous structure of nanomaterials provides excellent immobilization for enzymes, thereby effectively increasing the diffusion of both the target and electrolyte, advancing the catalysis for the analyte (Wen and Eychmüller, 2016). These nanomaterial sensing properties improve the performance and design strategies of wearable electrochemical biosensors. Conductive nanomaterials, particularly polymers, stand out as clear frontrunners, with significant advantages in explicit contact surface area, filler content, and operation electron transfer ratio. (Gangopadhyay and De, 2000). Polymers' versatility allows them to be synthesized in different forms, comprising elastomers, gels, and liquid crystal polymers (Dierking, 2010; Li et al., 2019), enlarging the morphology of wearable sensors to patch-like sensors, even micrometres thick, and tattoo-based electrochemical biosensors (Dierking, 2010; Li et al., 2019; Yoon et al., 2019). Electrochemical biosensors, together with immunosensors and DNA biosensors, are rapidly becoming the norm of the day (Kim et al., 2019). Among the numerous transduction systems used, electrochemical immunosensors have sparked the interest of researchers due to benefits such as a good detection limit, ease of automation, low cost, uniformity, and incorporation with miniaturized readouts, and comprehensive compatibility for onsite testing. Their sensing technologies and detection range are frequently improving because of advancements in the distinctive properties of conductive nanomaterials, particularly conductivity and electrochemical activity (Shaikh et al., 2019). Intercalation of interactional fillers into nanomaterials matrices improves the stability of functional electron transfer sites and identification limits, which has an influence on sensing applications. These efficient fillers aid in reducing layer stiffness in nanomaterials, paving the way for ultrathin electrochemical detector technology (Huang and Kaner, 2004; Zhou et al., 2014; Bandodkar et al., 2015a). In this review we have discussed the most recent research on electrode materials based on conductive nanomaterials and mechanized technologies for various types of wearable electrochemical biosensors.

We start with a brief overview of the basic design principle, and components of electrochemical biosensors. Following that, the next section provides a thorough explanation of the analytical applications of conductive nanoparticles in electrochemical biosensing. We discussed essential parameters for developing low-cost, sensitive, and porous sensing platforms with different technologies. Nanomaterials, polymers, and biological receptors create extremely sensitive and selective electrochemical sensing devices for electrode modification. Using other nanomaterials, MXene and composite materials such as conductive polymers in combination with CNT, Graphene, and metals that offer more sensor sensitivity are also addressed. Furthermore, a range of similar applications, such as the manufacture of biosensors, including immunosensor and DNA biosensors, in which conductive nanomaterials play a vital role in sensing performance, have also been investigated. The final section looks into the prospects and challenges of these wearable sensor systems' durability, robustness, and performance.

**GRAPHICAL ABSTRACT:**
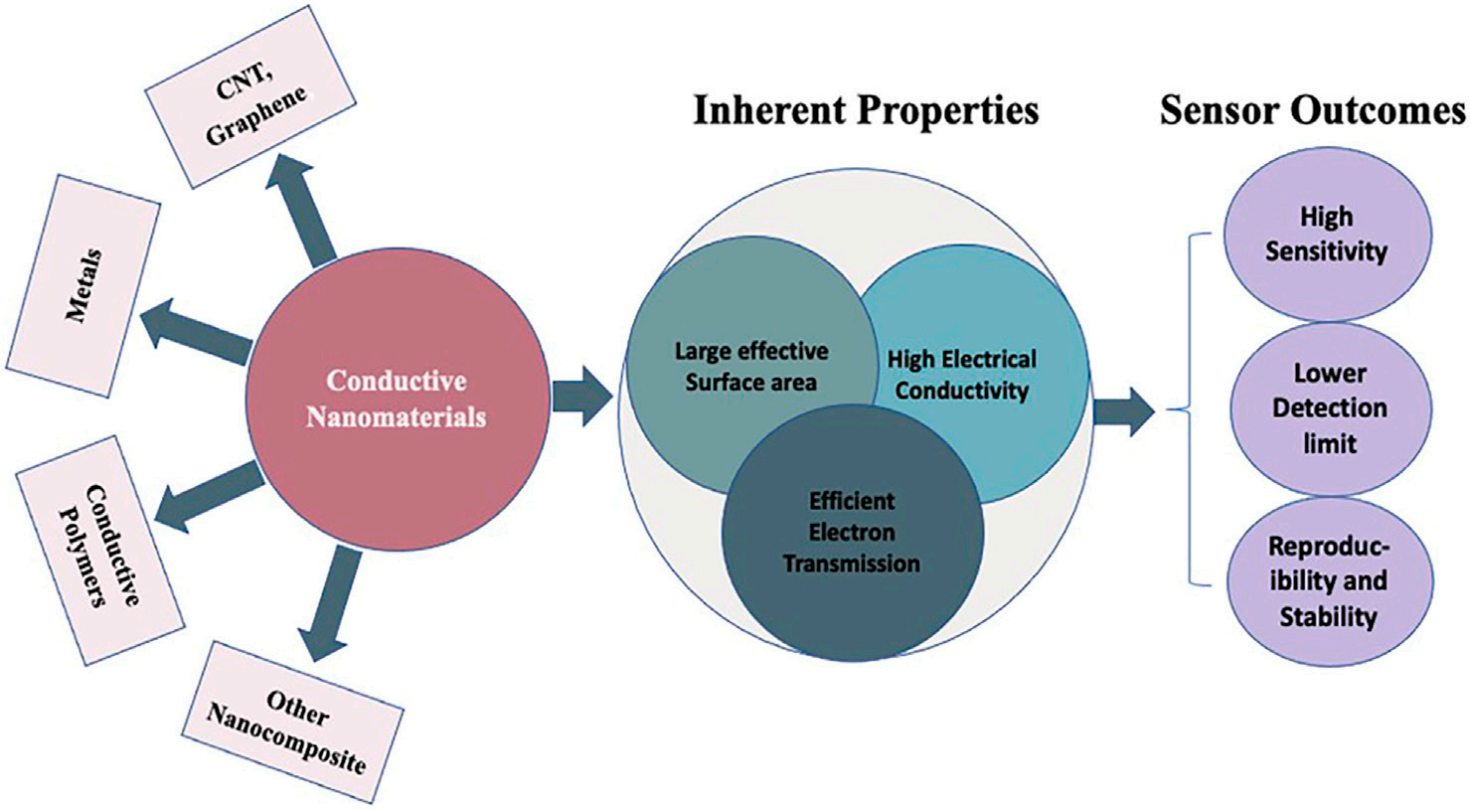


## Basic Design Principle and Component of Electrochemical Biosensor

**FIGURE 1:**
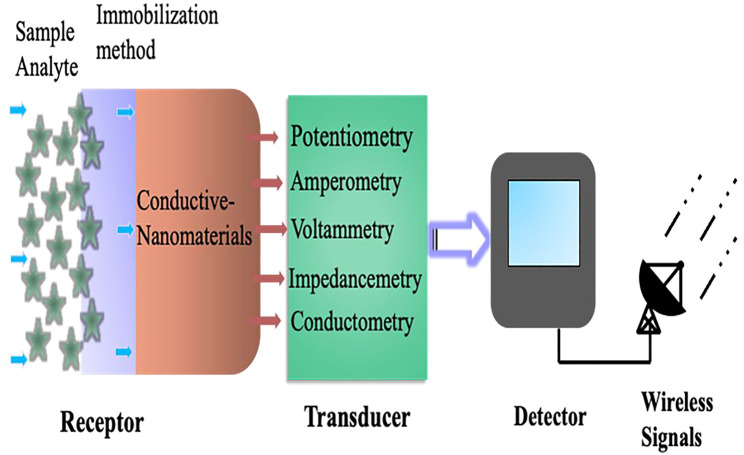
A schematic representation of the basic design principle and key components of an electrochemical biosensor.

Electrochemical biosensors work on the principle that an electrical current passes through a sensing electrode produced by an electrochemical reaction (the reaction between the electrode and analyte) that converts the associated information into qualitative or quantitative signals (Curto et al., 2012). In general, the reaction found between the electrodes is the result of electrical and chemical interaction. To obtain valuable information such as the concentration of a single entity in a sample, a molecular chemical receptor and a physicochemical detector component “transducer” are used. The transducer converts the chemical information into the analytical signals of the sample. Which eventually based upon the potentiometry, conductometry, and amperometric/voltammetric measurements. Table 1 summarizes a comparison of these various measurements with advantages and disadvantages. So, when a biological component like antibody, DNA, enzyme and ionophores is used in the recognition/receptor system, the device is referred to as a biosensor. The bioreceptor chosen for the chemical recognition system is analyte dependent and capable of producing analyte concentration in the form of physical or chemical signals with accurate and well-specified sensitivity (Pérez López, 2009) (Figure 1). Otherwise, modifying the working electrode with an ion-specific inert allows the sensor to detect specific electrolytes such as sodium, potassium, and calcium, etc. The changes in the flow of current could be seen depending on the concentration of specific metabolites such as glucose, lactate, urea, and other interstitial fluids. The sensor itself can be fabricated with flexible substrates like PDMS, Ecoflex, and textiles (Windmiller and Wang, 2013; Abellán-Llobregat et al., 2017), stretchable elastomers, tattoo papers, or some other plastic based fabric (Bandodkar et al., 2015a; Gao et al., 2016a; Nyein et al., 2016). Polyethylene terephthalate (PET) and polyimide (PI) are also commonly used substrates. Silk fibroin, cellulose, and sponge have recently gained a lot of attention because of their excellent biocompatibility and biodegradability (Bandodkar et al., 2015c; Bandodkar et al., 2016).

**TABLE 1:** Comparison of different sensing methods.

Method	Overview	Advantages	Disadvantages	Ref.
Potentiometry	The potential between the active and reference electrodes is measured at a constant current to detect the target analyte, such as the ion concentration.	Signal detection and signal handling are simple and systematic. Superb for charged species with a predetermined charge condition. Excellent for moderately concerted species, particularly in the mM range.	Applicable only to charged species sensing. Since this approach compares action to concentration, a selective membrane layer to target individual ions must be suggested. Contamination from other charges is a big concern for low-concentration ions.	Zdrachek and Bakker (2020)
Amperometry	It measures the current produced at a constant applied potential during the redox reaction that is proportional to the target analyte concentration.	Simple recognition and simple post-processing are needed to change current to concentration. Mediators may be used to reduce the necessary potential and hence power usage.	The Faradaic signal can fall off over time for traces of species below the “μM” scale, resulting in incorrect concentration conversions. An enzyme normally provides selectivity.	Borgmann et al. (2011)
Voltammetry	A voltage scan between the active and reference electrodes is performed, and the current properties are obtained to determine the concentration.	Since various species have different oxidation and reduction potentials, a voltage scan on two identical electrodes will provide information on multiple analytes simultaneously. Therefore, there are several sub-techniques to choose from in order to maximize the signal-to-noise ratios. can be combined with pre-concentration procedures for the identification of trace molecules, resulting in increased limitability.	This method requires more complex postprocessing in order to extract and distinguish the peaks from the necessary analyte. Background reactions may be activated by voltage scans, interfering with the appropriate signals.	Chen and Shah (2013), Dheilly et al. (2008)
Conductometry	Determine the variation in biological element conductance that occurs between a pair of metallic electrodes.	They may not need the use of a reference electrode; they work at low-amplitude alternating voltage, which prevents Faraday processes on electrodes; They are light insensitive;	Despite the fact that electrochemical biosensors are particularly sensitive to hydrogen, their sensitivity decreases with time due to the rapid deterioration of the electrode catalyst, which in process applications is easily polluted by process gases.	Pungor (2016)

## Analytical Applications of Conductive Nanomaterials in Electrochemical Biosensors

### Carbon Nanomaterials for Wearable Electrochemical Biosensors

Carbon nanomaterials are the main building blocks in nanotechnology, which have attracted much attention because of their large specific surface area, high mechanical strength, inherent structural defects, good electrical conductivity, and excellent chemical and thermal stability (Park et al., 2013). Carbon nanotubes (CNTs), graphene (Grp) and reduced graphene oxide (rGO) are the most commonly used carbon nanomaterials in electrochemical biosensors. It appears to be a fantastic material, with a tensile strength of a hundred times that of steel, more excellent thermal conductivity than diamond, and electrical conductivity equivalent to copper but with the ability to carry a large amoiunt of current. In this section, analytical applications of carbon nanomaterials such as CNT, graphene and reduced graphene oxide have been demonstrated for wearable electrochemical biosensors.

#### Electrochemical Biosensors Based on “Carbon Nanotube”

**FIGURE 2:**
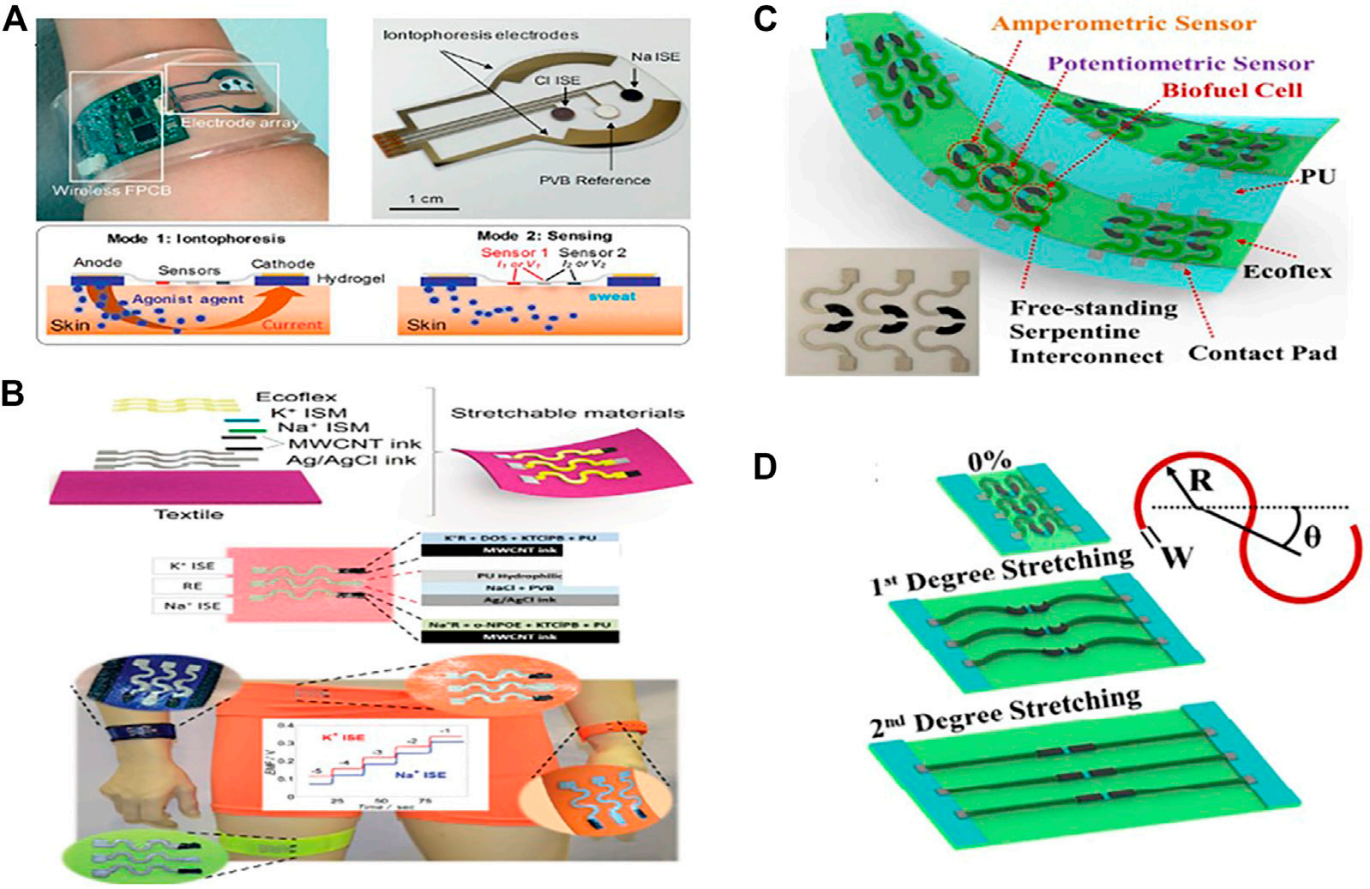
Electrochemical biosensors that can be worn. **(A)** Image of an independent sweat extraction and sensor platform implanted on the skin, with a small layer of agonist agent hydrogel inserted under the iontophoresis electrodes (Copyright 2017; PNAS). **(B)** A wearable and stretchy CNT-based sensor printed on several standard textiles, as well as normal potassium and sodium measurement time trace graphs (Copyright 2016; Advanced Healthcare Materials). **(C)** all-in-one printed electrochemical biosensor and biofuel cell (BFC) arrays (copyright 2015, Nano Letters). **(D)** The two degrees of stretching allow printed arrays to withstand high strain levels (Copyright 2015; Nano Letters).

Carbon nanotubes (CNTs), as 1-D nanomaterial, have recently gained a lot of attention as a valuable material for developing wearable electrochemical biosensors, particularly amperometry and potentiometric pH sensors, as some of them are listed in Table 2. CNTs are fabricated onto electrochemical transducers in different ways, mostly by coating and printing electrode substrates with CNTs or onto the composite electrodes (Park et al., 2013). Analyzing sweat from the human body during exercise may provide valuable information by monitoring the levels of electrolytes, e.g., (pH, Na^+^, K^+^, and Ca^+2)^ and metabolites (glucose, lactate, urea) as well as skin interstitial fluid (Schazmann et al., 2010;Bandodkar and Wang, 2014). Sam Emaminejad et al. have reported a wearable electrochemical biosensor based on CNT for glucose measurement in human sweat (Figure 2A). The fabricated wearable sensor generates current signals proportional to the glucose concentration in a linear range of 0–100 mol/L with a sensitivity of 2.1 nA L m/mol, proving the excellent performances of the proposed glucose sensor, which is fabricated in such a way that CNTs is used as Glucose oxides (GOD’s) immobilization matrix as well as H_2_O_2_ sensing components. The concentration of glucose will be determined indirectly by detecting H_2_O_2_ (Emaminejad et al., 2017). Wang and his colleagues improved wearable technology combined with electrochemistry, enabling the development of novel new sensing platforms for non-invasive on-body and on-site applications in sports, exercise, and healthcare. His group, for example, proposed a CNT-printed textile-based potentiometric pH sensor capable of sensing electrolytes such as sodium and potassium in human sweat (Parrilla et al., 2019). Stretchable components such as (CNT and Ag/AgCl resistive inks) and pu ecoflex were printed in a serpentine pattern array on a textile substrate and then modified with ion-selective membranes (Figure 2B) (Parrilla et al., 2016). The sensor is capable of withstanding high tensile stress without cracking. Using open circuit potential readings, the sensor was checked. A calibration curve was recorded between the change in EMF and the time and by adjusting the concentration of NaCl and KCl solutions. The electrocatalytic response for the sodium [Na+] selective electrode was 59.4 mV log^−1^ for a linear range of 10^–4^ to 10^–1^ m with a detection limit of 104.9 M and the potassium [K^+^] selective electrode had an electrocatalytic response of 56.5 mV log^−1^ over a linear range of 10^–4^ to 10^–1^ m with a detection limit of 104.9 M. While various types of electrochemical biosensors with various functionalities, such as multifunctional and wireless, have been established, there is still a big challenge in achieving self-powered electronics and energy harvesting techniques in electrochemical sensing.

**TABLE 2:** Carbon based nanomaterials in electrochemical biosensors.

Sensing material	Bio-fluid	Analyte	Detection range	Method	Ref.
Bare carbon	Sweat	b-nicotinamide adenine dinucleotide	0–3 mM ferrocyanide, 0–25 mM hydrogen peroxide, 0–100 μM NADH	Amperometry	Yang et al. (2010)
Bare carbon	Sweat	Uric acid	-	Voltammetry	Windmiller et al. (2012)
CNTs	Sweat	pH, K^+^, NH_4_	pH 8.51 to 2.69	Potentiometry	Guinovart et al. (2013)
Prussian blue (PB) onto CNT fibers	Sweat	Glucose	2.15 nA µM^−1^	Amperometry	Wang et al. (2018)
Graphene doped Au mesh	Sweat	Glucose, pH	10 × 10^–6 m^ (glucose)	Potentiometry	Lee et al. (2016)
CNT	Sweat	Glucose, lactate	0.3 × 10^–3 m^ (lactate)	Amperometry	Jeerapan et al. (2016)
CNT	Sweat	NH4^+^, Glucose	-	Amperometry	Bandodkar et al. (2016)
CNT	Sweat	Glucose, lactate,	-	Amperometry	Gao et al. (2016a)
		Na^+^, K^+^			
Carbon/rGO	Sweat	Na^+^, K^+^, pH,	10–160 mM	Potentiometry	Xu et al. (2019)
			2–32 mM		
			3–8		

Wang and his colleagues developed for the first time an all-in-one printed CNT-based electrochemical biosensor and biofuel cell (BFC) array (Figure 2C). A framework based on CNT functionalized with selective ionophores and enzymes was designed to understand various applications. Such as amperometric enzyme-based glucose sensors, potentiometric ammonium sensors, self-powered biosensors, and enzymatic glucose biofuel cells (BFCs). It can endure up to 500% strains without losing structural stability or sensor performance (Bandodkar et al., 2016)**.** The electrochemical characterization of the system revealed that repeated strains ranging from 0 to 500%, torsional twisting of 180° for 50 cycles, and indenting stress (5 mm depth for 50 repetitions) has no impact on its device properties (Figure 2D). Another CNT-silver nanoink-based BFC with a textile substrate has been used as a self-powered sensor ability to extract perspiration energy and inspect sweat metabolites such as glucose and lactate (Jeerapan et al., 2016). Further, CNTs can be classified into two types: single-wall carbon nanotubes (SWCNTs) and multi-wall carbon nanotubes (MWCNTs) (Willner et al., 1996). Marc Parrilla et al. recently formed MWCNTs-based wearable potentiometric ion sensors (WPISs) to measure pH and ions (Na+, K+, and Cl−) in human sweat during exercise or exercise some other kind of sports. The sensors have Nernstian slopes within the approximate physiological range of each ion analyte, such as (for Na^+^: 10–100 mM, K^+^: 10–10 mM, Cl^−^ : 10–100 mM, and pH range: 4.5–7.5) (Parrilla et al., 2019).

#### Electrochemical Biosensors Based on Graphene

**FIGURE 3:**
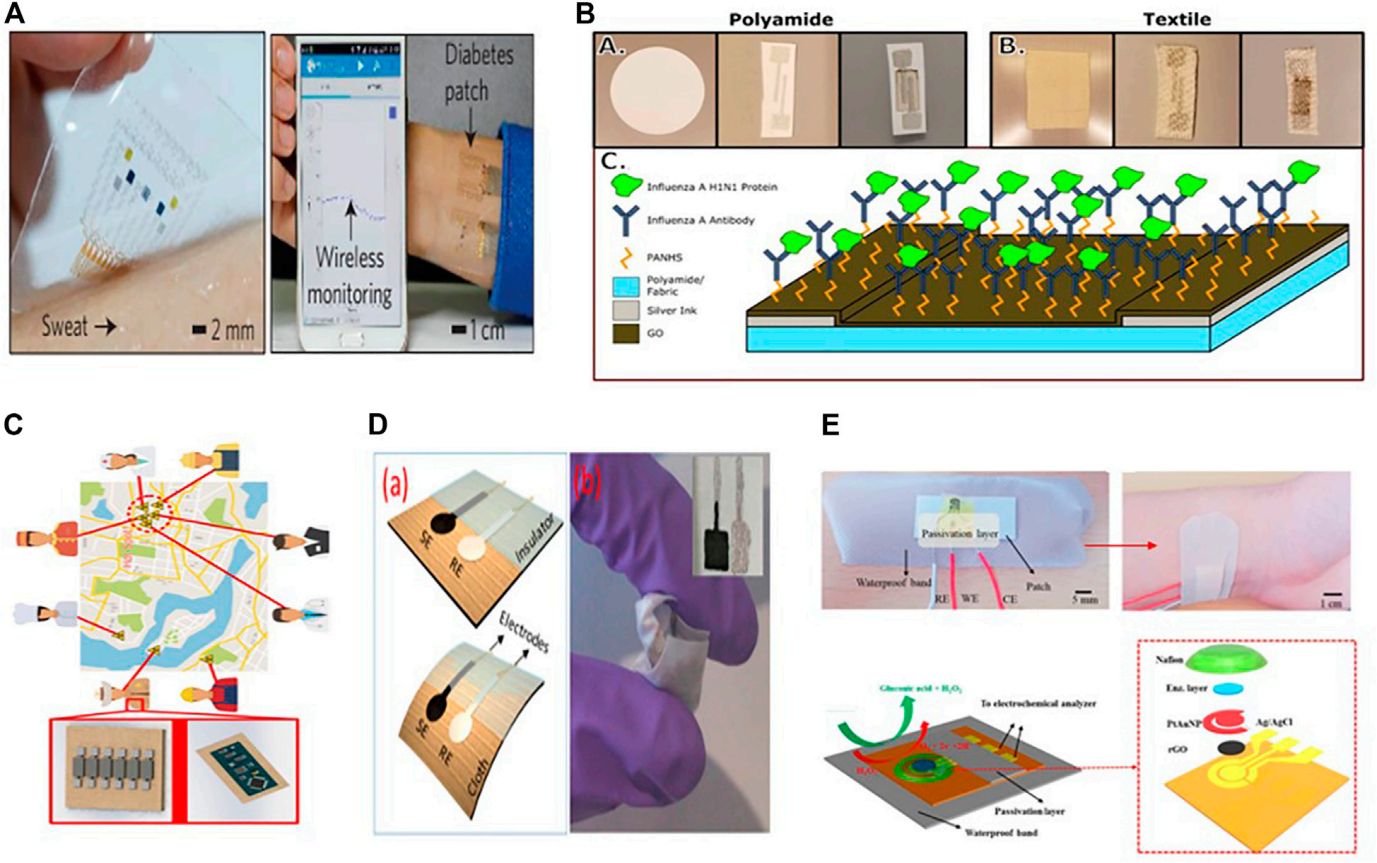
**(A)** An optical image of a graphene-based hybrid electrochemical device mounted on human skin for automatic diabetes monitoring (Copyright 2016; Nature Technology). **(B)** A screen-printed graphene oxide-based biosensor using graphene oxide transduction film on both nanoporous polyamide and textiles substrates for tracking environmental exposure to influenza a virus (Copyright 2018; Electrochemical Society). **(C)** Schematic diagram of environmental exposure to influenza a virus (Copyright 2018; Electrochemical Society). **(D)** A lightweight potentiometric pH sensor, SE-sensitive electrode, and reference electrode are schematically represented on cloth (Copyright 2019; Biosensor). **(E)** Photographs and schematic representation of the completed sweat-based glucose biosensor mounted on human skin (Copyright 2018; Biosensors and Bioelectronics).

Graphene has become a leading material due to its outstanding properties, such as high charge carrier immovability, chemical stability in aqueous conditions, large effective surface area, and the ability to have a porous 3-dimensional structure. Graphene, on the other hand, can effectively increase the toughness and stretchability of electrodes. Because of its superior mechanical properties and high flexibility, making it more appropriate for use in wearable electrochemical biosensors (Shan et al., 2009; Lin et al., 2020; Zhang et al., 2021), as some of them are listed in Table 2. Lee et al. proposed a wearable sweat based glucose sensor using 2-D graphene nanomaterial modified with glucose oxides (Lee et al., 2016). Sweat concentration in glucose was accurately measured to assess the glucose levels (Figure 3A). Due to the high flexibility and good mechanical strength of graphene, the sensitivity of the proposed glucose sensor was maintained well under large stress. In addition, a gold doped graphene and gold mesh render compatible wearable patch with 30% stretchability was also presented for the measurement of metabolite (glucose, lactate) in (10 × 10^–6^ to 0.7 × 10^–3^ m) range (Lee et al., 2016). Wang et al. introduce a different sensing electrode made of graphene oxide paper and modified with Cu_3_(btc)_2_ nanotubes and stable amino designed to detect glucose in sweat. This sensing electrode was used for a non-enzymatic electrochemical platform. The built wearable sensor has incredible sensitivity because of the large specific surface area caused by the graphene porous structure.

After all, David et al. developed a textile screen-printed graphene oxide-based biosensor. They used conductive silver ink electrode and graphene oxide transduction film on both nanoporous polyamide and textiles for everyday use to track environmental exposure to the influenza A virus (Figure 3B) (Kinnamon et al., 2018). An influenza-specific affinity assay was developed using electrochemical impedance spectroscopy (EIS) to identify the virus in solution on this graphene oxide layer because it is more secure and repeatable on the textile substrate than polyamide. Since the textiles sensor has high detection capabilities, it has a linear dynamic range of 10 ng/ml to 10 g/ml and a maximum 10 ng/ml detection in the biological fluid equivalent (buffer). The sensor demonstrates the ability to be integrated with the internet of things (IoT) devices so that it can wirelessly detect flu detection, helping to build a space-specific heat map of virus contacts (Figure 3C), which could be helpful for medical personnel identification and to stop the virus outbreak before it spreads out.

For additional biological applications (Zamora et al., 2018), P. Salvo et al. also demonstrated a potentiometric pH sensor based on a graphene sensitive layer with 40 mV/pH sensitivity in the pH range of 4–10 (Figure 3D) (Salvo et al., 2018). Within a week, they tested five sensor prototypes in human serum samples. The average deviation of the average response from the reference value collected by the glass electrode was 0.2 pH units. Recently Manjakkal Libu et al. also reported a graphene-based potentiometric pH sensor for wearable health tracking applications on a textile substrate. A thick G-PU film as a sensitive electrode (SE) was printed on cellulose-polyester blend fabric. The sensor shows a sensitivity of 4 mV/pH and a reaction time of 5 s in the pH range of 6–9. After washing in tap water, the sensor’s performance is almost a potential 47 ± 2 mV for a long time (2000 s) (Manjakkal et al., 2019). For wireless monitoring of respiration and bacteria, a graphene printed silk sensor was fabricated and applied to tooth enamel and then functionalized with anti-microbial peptides to actively recognize “Helicobacter pylori” cells in saliva (Mannoor et al., 2012). Notably, in wireless operating mode, the sensor achieved a measurement standard of one bacterium μl^−1^ for a range of 103–108 CFU ml^−1^.

#### Reduced Graphene Oxide Based Electrochemical Biosensor

Reduced graphene oxide is an excellent application material for electrochemical biosensor data processing. By easily incorporating functional groups and easy synthesis to parental chain, it has emerged as a viable alternative to other composites. Compared to other sensors on the market, rGO-based electrochemical biosensors provide high stability at lower temperatures without considering humidity. This element of low graphene oxide is likely to be investigated further in low-temperature sensors.

Xuan et al. developed a reduced graphene oxide (rGO) nanocomposite based electrochemical sensor to monitor body sweat measurement. The sensor was successfully fabricated on a flexible polyimide substrate using a simple and low-cost fabrication method. Gold Platinum nanoparticle alloy was deposited onto the (rGO) modified working electrode (Figure 3E), the sensor worked well in analytical operation (Xuan et al., 2018).

### Metal-Based Nanomaterials for Wearable Electrochemical Biosensors

As electrochemical sensing materials, metal and metal-oxide based nanoparticles have attracted a lot of attention because of their small size, outstanding mechanical, electrical, chemical properties and high catalytic efficiency, as well as their versatility in creating new and better sensing systems (Bhide et al., 2019; Shaikh et al., 2019; Li et al., 2020). they can be categorized into noble and non-noble metal-based nanomaterials. Rh, Ir, Pt, Ru, Au, Os, and Ag are good examples of noble metal nanoparticles (Imamura et al., 2020). As it has been described earlier, metal-based nanomaterials have excellent and promising electro-catalytic properties, especially in wearable glucose non-enzymatic sensors. Like platinum Pt, palladium Pd, gold Au, metallic and oxides such as CuO, NiO, which can directly catalyse glucose (Abellán-Llobregat et al., 2017; Toi et al., 2019; Li and Wen, 2020), some metals based electrochemical biosensors are highlighted in Table 3.

**TABLE 3:** Metal based nanomaterials in electrochemical biosensors.

Sensing material	Bio-fluid	Analyte	Detection range	Method	Ref.
ZnO	Body fluid	Pesticide	-	Potentiometric	Hatamie et al. (2015)
(Ag/AgCl)	Sweat	Glycemic	-	Amperometry	Bandodkar et al. (2015b)
Platinum	Sweat	Oxygen	(11 s–90% of a steady-state current)	Amperometry	Mitsubayashi et al. (2003)
Au, Bi	Sweat	Zn	10.4 nAL µg^−1^	Voltammetry	Gao et al. (2016b)
NiCo_2_O_4_/chitosan	Sweat	Glucose	0.5 μA/μM	Amperometry	Lu et al. (2019)
Bi	Sweat and urine	Cd^2+^	<100 μg L^−1^	Voltammetry	Gao et al. (2016b)
Bi, Au	Sweat and urine	Pb^2+^	<100 μg L^−1^	Voltammetry	Gao et al. (2016a)
Au	Sweat and urine	Cu^2+^	100–1,000 μg l–1	Voltammetry	Gao et al. (2016a)
Au	Sweat and urine	Hg^+^	<100 μg L^−1^	Voltammetry	Gao et al. (2016a)
Au	Sweat	Glucose	0–200 μM	Amperometry	Gao et al. (2016a)
		Lactate	0–30 mM		
Ag/AgCl	Sweat	Chloride	-	Potentiometric	Gonzalo-Ruiz et al. (2009)
Bare gold	Tears	Electrolytes	-	Conductometry	Ogasawara et al. (1996)
Ag/GOx	On body	Bio fluid influenza A virus	LDR: 10 ng ml^−1^ to 10 μg/ml LOD: 10 ng ml^−1^	Potentiometry	Kinnamon et al. (2018)
Graphite/Ag/AgCl	Sweat	pH	pH range 6–9	Potentiometry	Manjakkal et al. (2019)
Vertically aligned gold nanowires	Sweat	Na^+^, K^+^	(56.1 mV/pH for pH, 58.2 mV/decade for Na^+^ and 41.5 mV/decade for K^+^)	Potentiometry	Wang et al. (2020)
Platinum-decorated graphite	Sweat	Glucose	33 μM and 0.9 mM	Amperometry	Abellán-Llobregat et al. (2017)

**FIGURE 4:**
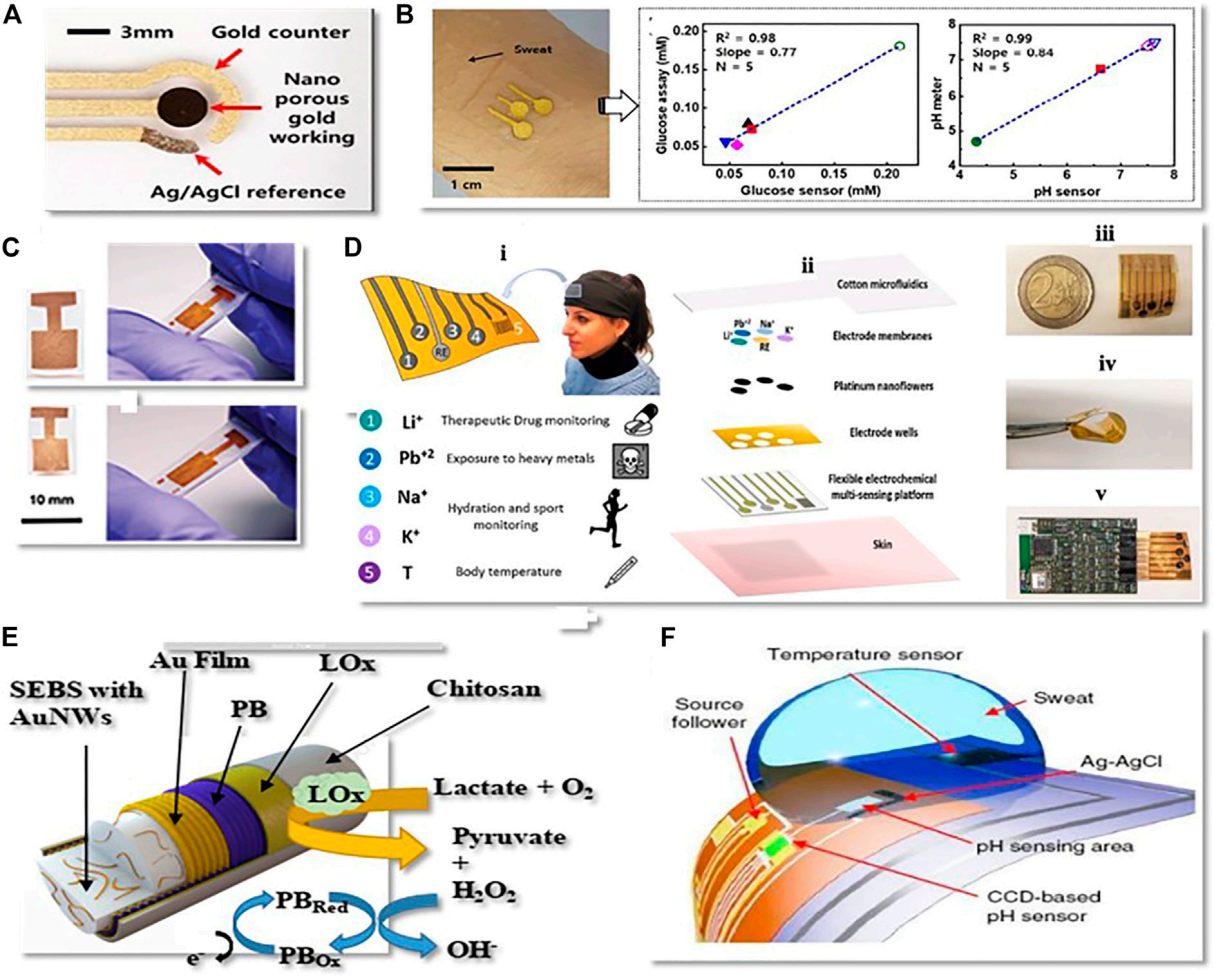
**(A)** Gold nanoporous based non-enzymatic wearable biochemical sensor (Copyright 2019; American Chemical Society). **(B)** Gold nanosheet-based non-enzymatic wearable biochemical sensor (Copyright 2018; American Chemical Society). **(C)** Unshrunk gold electrode on polyolefin (PO) and shrunk electrode on elastomer substrate (Copyright 2020; Advance Material and Technology). **(D)** Overview of the wearable multi-electrode device for sweat-based healthcare surveillance (Copyright 2020; Sensors and Actuators): (I) testation of health care applications; (II) flexible electrochemical multi-sensing system; II) flexible multi-electrode sensing platform; (IV) bending test; (V) example of interfacing with read-out electronics. **(E)** Lactate tracking electrochemical biosensor based on gold nanofibers. (Copyright 2021; Journal of Materials Chemistry). **(F)** Ultrasensitive potentiometric biosensors based on Quasi-two-dimensional metal oxide semiconductors (Copyright 2018; Springer Nature).

Typically, glucose’s sensing mechanism consists of non-noble metal-based nanomaterials by the redox reaction of the hydroxyl (−OH) group (Archana et al., 2019). However, metal-based nanomaterials can be used as nanowires or nanosheets to achieve maximum sensitivity; that’s why some researchers use metal nanosheets and nanowires to achieve the maximum sensitivity instead of making film electrodes as shown in (Figures 4A,B) (Wen et al., 2014;Oh et al., 2018;Bae et al., 2019). In the last few years, gold has become a common active sensing material because of its excellent biocompatibility, and electrochemical property. Amanda et al. made a thin-film gold electrode-based glucose sensor (Imamura et al., 2020). The stretchable electrodes stretchability is almost 210% of its original length and accurately detect the glucose level without enzymes, which is one of the lowest documented for flexible, enzyme-free sensor (Figure 4C). Nowadays, metallic aerogels, a new category of 3-D metal-based nanomaterials anticipated by researchers, have recently become a new field of concern, offering enormous glucose bio-electrocatalysis and promise in wearable glucose sensors (Wen et al., 2016). Its porous form and gel state are ideal for immobilization of enzymes and maintaining their activity, which is highly beneficial in extending the life span of wearable glucose sensors (Zhang et al., 2012). Wen et al. developed a glucose sensor by assembling three-dimensional gold aerogels nanostructure modified glucose oxides. The sensor can detect glucose qualitatively and quantitatively in 0.1 mol/L phosphate buffer solution (pH 7.4) (Wen et al., 2016). In addition, compared with a single metal system, a reasonably designed multi-metal nanomaterial can make extensive use of the properties of two or more metal elements. thus enhancing the efficiency of the wearable glucose sensor, which will also become the metal sensor’s production path. Wang et al. and his colleagues recently created elastomer-bonded gold nanowire coating technology. Using the same technology, they create lactate-sensing working electrodes, reference electrodes, and counter electrodes for lactate monitoring in human sweat (Figure 4D).

In artificial sweat, the sensor has a high sensitivity of 14.6 μA/mM.cm^2^. This sensor’s sensitivity is maintained even when subjected to high tensile strains of up to 100% without the use of any external structural layout (Wang et al., 2020). Their group has also suggested a gold fibre-based wearable electrochemical biosensor for sweat PH tracking. The manufactured fibre-based pH sensor shows superior sensitivity (60 mV/pH), high selectivity against cationic interference, and high stretchability (up to 100% strain). One benefit of fibre-based sensors is their ability to be incorporated in textiles, which can be integrate into daily garments to detect “unrecognizable” personal health (Wang et al., 2020). Recently, Francesca et al. designed a wearable multifunctional sweat sensing system based on platinum. The device is highly flexible and comprises four electrodes for continuous measurement of analytes such as Li^+^, Pb^+2^, K^+^ and Na^+^ in different health applications and sports activities (Figure 4E). The sensors show linear responses in artificial sweat. Because of the good biocompatibility, flexibility and accurate sample handling this wearable framework is a significant step forward in the advancement of non-invasive tracking technologies for health, opening the way for better understanding of physiological parameters and clinical needs of individuals (Criscuolo et al., 2021). Metals containing oxides, such as indium oxide and lead oxide, are more sensitive and can be used to make sensing electrodes for wearable electrochemical biosensors. Huajun et al. suggested a pH sensor based on quasi-two-dimensional metal oxide semiconductors for detecting glucose and ph in sweat. The sensor is made of In_2_O_3_ thin films and has a detection limit of 0.0005 for pH and high accuracy in detecting glucose content (Figure 4F) (Chen et al., 2017).

### Conductive Polymer Nanomaterials Based Electrochemical Biosensors

**FIGURE 5:**
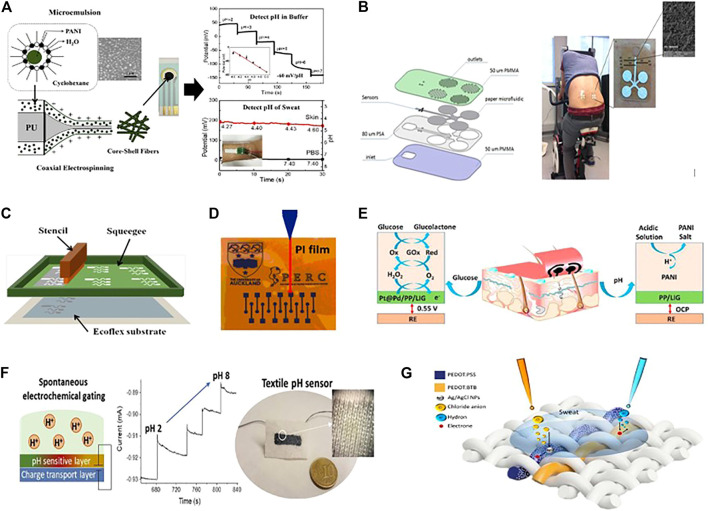
**(A)** Fiber coaxial electrospinning with graph monitor, pH determination in buffer, and sweat (Copyright 2020; Polymers & Biopolymers). **(B)** A schematic representation of the microfluidic chip fabrication steps is seen, as well as a photograph of the platform attached to the body and an image from scanning electron microscopy (SEM) (Copyright 2017; Biosensors and Bioelectronics). **(C)** Screen-printed stretchable device with custom stretch-resistant (Copyright 2015; Wiley). **(D)** Laser writing using a CO_2_ laser. (Copyright 2018; Biosensors and Bioelectronics). **(E)** A wearable glucose sensor based on poly (3, 4-ethylene dioxythiophene)-poly (styrene sulfonate) (Copyright 2020; Elsevier). **(F)** PH sensor with two terminals wearable sensor and magnification of cloth knotwork Current vs time reaction to pH changes (Copyright 2017; Biosensors and Bioelectronics). **(G)** Conductive Polymers based textile chemical sensor for Sweat analysis (Copyright 2020; Scientific Reports).

Polymer-based novel sensing capabilities represent a significant advancement in electrochemical sensing. Since the electrochemical sensors are integrated into textile structures through weaving, knitting, and embroidery, or need to be directly embedded into garments and coupled at the human skin’s surface to detect the target analyte (Allison et al., 2017). It is highly desirable that the sensors should be reliable and flexible enough to reduce motion-induced signal interference. While carbon and metal-based nanomaterials are highly sensitive but they are not flexible enough as the conductive polymer-based nanomaterials. It is well recognized that CPs have advantages such as chemical diversity, low density, durability, corrosion resistance, easy-to-handle shape, terminology and adaptable conductivity. Moreover, the outstanding properties of flexible conductive polymer nanomaterials are as follows: 1) they aided in increasing the selectivity and stability of electrochemical biosensors' sensing properties. 2) They usually have a 3-D structure. They can be manufacture in various sizes, including nanometre size and high conductivity, such as 120–130 S cm^−1^ at room temperature, which offers a more precise surface area. 3) The modification of conductive polymers at the sensing electrodes is relatively very easy and simple because it has no special requirements for the evenness of electrodes (Matsumura et al., 2018) (Gerard et al., 2002;Gerard and Malhotra, 2005;Zeng et al., 2014). Researchers discovered and produced conductive polymer nanomaterials such as (PEDOT- PSS) (Abouraddy et al., 2007;Zhao et al., 2018), polypyrrole (ppy) (Gregory et al., 1989;Tzou and Gregory, 1992)), polythiophene (PTh) and polyaniline (PANI) (Huang and Kaner, 2004;Teli et al., 2014), to fabricate the sensing electrodes. Usually, conductive polymers are deposite at working electrodes as a compact film. Some conductive polymer nanomaterial-based wearable electrochemical biosensors are summarized in Table 4. Xuesong et al. developed PANI nanoparticle-based sensitive pH sensor. The sensor was fabricated by coaxial electrospinning of PANI nanoparticles and polyurethane (PU) into the core-shell fibres (Figure 5A). A Screen-printing method was used to create a sensing electrode assembly on a polyethylene terephthalate (PET) substrate, which consisting of gold as a counter electrode, (Ag/AgCl) reference electrode, and (PANI-PU) working electrode. Here, PU provides mechanical stability to the sensor. The feasibility of detecting sweat pH on the skin was demonstrated by attaching the chip to the arm and electrochemical workstation was used to measure the reaction. The sensor operated linearly in the pH range of 2–7 with a sensitivity of 60 mV/pH and can detect pH changes of less than 0.2 (Hou et al., 2020). Salzitsa et al. developed a fully wearable and flexible patch with completely integrated sensing system for on body human sweat testing (Figure 5B).

**TABLE 4:** Conductive polymer nanomaterials in electrochemical biosensors.

Sensing material	Bio-fluid	Analyte	Detection Range	Method	Ref.
PANi conducting polymer	wounds	pH	pH range (5.5–8)	Potentiometric	Guinovart et al. (2014)
(PEDOT:PSS)	Sweat	Na^+^	45.8 mV dec^−1^	Potentiometry	Wang et al. (2018)
(PEDOT:PSS)	Sweat	K^+^	35.9 mV dec^−1^	Potentiometry	Wang et al. (2018)
(PEDOT:PSS)	Sweat	Ca^2+^	52.3 mV dec^−1^	Potentiometry	Wang et al. (2018)
Polyaniline (PANI) onto the CNT fibers	Sweat	pH	-	Potentiometry	Wang et al. (2018)
PEDOT:PSS/Ag/AgCl	Sweat	Cortisol	59.63 mV pH^−1^	Amperometry	Parlak et al. (2018)
PEDOT/RGO/GC	Rat brain	Dopamine	0.1–175 μM	Amperometry	Xu et al. (2014b)
Polyaniline	Sweat	pH	3–8	Potentiometry	Nyein et al. (2016)
PEDOT	Sweat	Na^+^ K^+^	-	Potentiometry	Lee et al. (2016)

The sensing device is designed so that a steady stream of sweat can flow through an array of compact microneedles with a diameter of 50 μm. The sensors are embedded in a microfluidic channel, which can simultaneously track metabolites (lactate) and electrolytes, for example, pH and sodium ions. The promise of the multi-sensing platform for monitoring the metabolite and electrolyte (sodium, lactate, and cortisol) from saliva is demonstrated in detail. For that, a potentiometric sodium ion sensor made of poly (3, 4-ethylenedioxythiophene) (PEDOT) and an amperometry lactate sensor were proposed. This sensing device can transmit data wirelessly for easy processing and storage, with the potential for real-time data analytics (Anastasova et al., 2017). As it has been seen, various groups have achieved device dimensional stability through lithographic or coating processes that are either costly or complicated on a large scale. So, for the first time, AJ Bandodkar et al. filled this technological gap by using the screen-printing method to create a low-cost and highly stretchable (PEDOT: PSS) based wearable electrochemical biosensor (Figure 5C). The stretchable device exhibits 2-D serpentine interconnects with 180° turns between electrode areas and touchpads (Bandodkar et al., 2015c). This electrochemical device has high sensitivity, can undergo high tensile stress, and meets the stretchability criteria of many applications; it was a good sign for potential and next-generation wearable systems. Tomas Guinevere et al. also developed a screen printed Potentiometric pH sensor based on conductive polymer (PANI) to measure the pH level of wounds in the body (Guinovart et al., 2014). The device works by carefully incorporating a pH sensor into bandages (pH range 5.5–8). These new pH-sensitive bandages opened a new possibility for the realization of telemedicine. In 2018, Xu et al. introduced a new and innovative technique to develop a highly sensitive PEDOT-based electrochemical biosensor for the detection of dopamine (DA) (Figure 5D) (Xu et al., 2018). The sensor was fabricated using a PEDOT-modified laser scribed graphene (LSG) method, which shows higher sensitivity and selectivity for detecting dopamine in a complex mixture. These PEDOT-LSG electrodes have a lot of potential for infield or point-of-care biosensing and some other incorporated bioelectronics products. Recently, Zehad et al. used the same technique to develop a PEDOT-PSS modified 3-D stable porous, porous, laser-induced graphene (LIG) to detect glucose and pH in human sweat (Zahed et al., 2020). Where, PEDOT-PSS is used to increase the tensile stability and uniform conductivity of the electrode. The fabricated electrochemical biosensors display a good current response to glucose in a wide linear range of 10 mmol/L to 9.2 mmol/L, with high sensitivity of 247.3 mA L mmol cm^−2^ and a low detection limit of 3 mmol/L. As a new kind of multifunctional sensor, this versatile substrate was further improved with Pt/Pd nanoparticles for glucose detection (Figure 5E). Recently Mariani et al. proposed a PEDOT: PSS film based electrochemical potentiometric sensor through a new and different approach. A pH-dependent modification of the current flowing through the PEDOT: PSS film was obtained through the random electrochemical gating caused by the potentiometric transducer (PEDOT: BTB), PEDOT: Bromothymol Blue [27]. The feasibility of this technique was demonstrated by creating a screen-printed pH sensor on a bio-ceramic fabric (Figure 5F). Compared to the rigid analogous fabricated on a glass substrate, this textile pH sensor demonstrated a standardized sensitivity of (7.5) x10^−3^ pH^−1^ in the range of 2–7, with no penalty of sensing efficiency. By using the same content PEDOT:PSS and PEDOT:BTB (Mariani et al., 2020). The same technique was used to develop a thread-based pH sensor, as shown in (Figure 5G). In conjunction with another thread-based sensor for multi-sensing network and chloride ion (Cl^−^) detection, the textile sensor could detect pH selectively during continuous recordings (Possanzini et al., 2020).

### Other Sensing Nanomaterials

In accordance with the sensing nanomaterials described above, various novel nanomaterials with good electrical conductivity, large specific surface area, and good biocompatibility are developing in the field of wearable electrochemical biosensors. For example, Polymer nanomaterials flexibility, conductivity, durability and long-term stability would be enhanced further after compound with Carbon and metal-based nanomaterials (Zhou and Shi, 2016; Jia et al., 2019) as some of them have been listed in Table 5.

**TABLE 5:** Other conducting nanomaterials in electrochemical biosensors.

Sensing material	Bio-fluid	Analyte	Detection range	Method	Ref.
CNTs/Ag/AgCl/PANI	Interstitial fluid	pH	59.63 mV pH^−1^	Potentiometry	Mpanza (2016)
CNTs/Ag/AgCl/PANI	Interstitial fluid	Glucose	-	Amperometry	Mpanza (2016)
polymers/SWNT-COOH	On body	Body order Volatile amine	Linear dynamic range LDR:50–1000 ppm	Conductometry	Seesaard et al. (2015)
PEDOT:PSS/Au	Sweat	Na^+^	10–160 mM	Potentiometry	Gao et al. (2016a)
		K^+^	1–32 mM		
PEDOT:PSS/Carbon	Sweat	Na^+^	0.1–100 mM	Potentiometry	Yoon et al. (2019)
fiber thread		K^+^	0.1–100 mM		
MXene	Sweat	Glucose and lactate	35.3 µA mm^−1^ cm^−2^ for glucose, and 11.4 µA mm^−1^ cm^−2^ for lactate	Amperometry	Lei et al. (2019)
rGO-PANI	Sweat/fluid	PH	75.09 nm/pH at pH 11.35	Potentiometry	Semwal and Gupta (2019)

#### Conductive Polymer Combines With Metallic Nanomaterials

**FIGURE 6:**
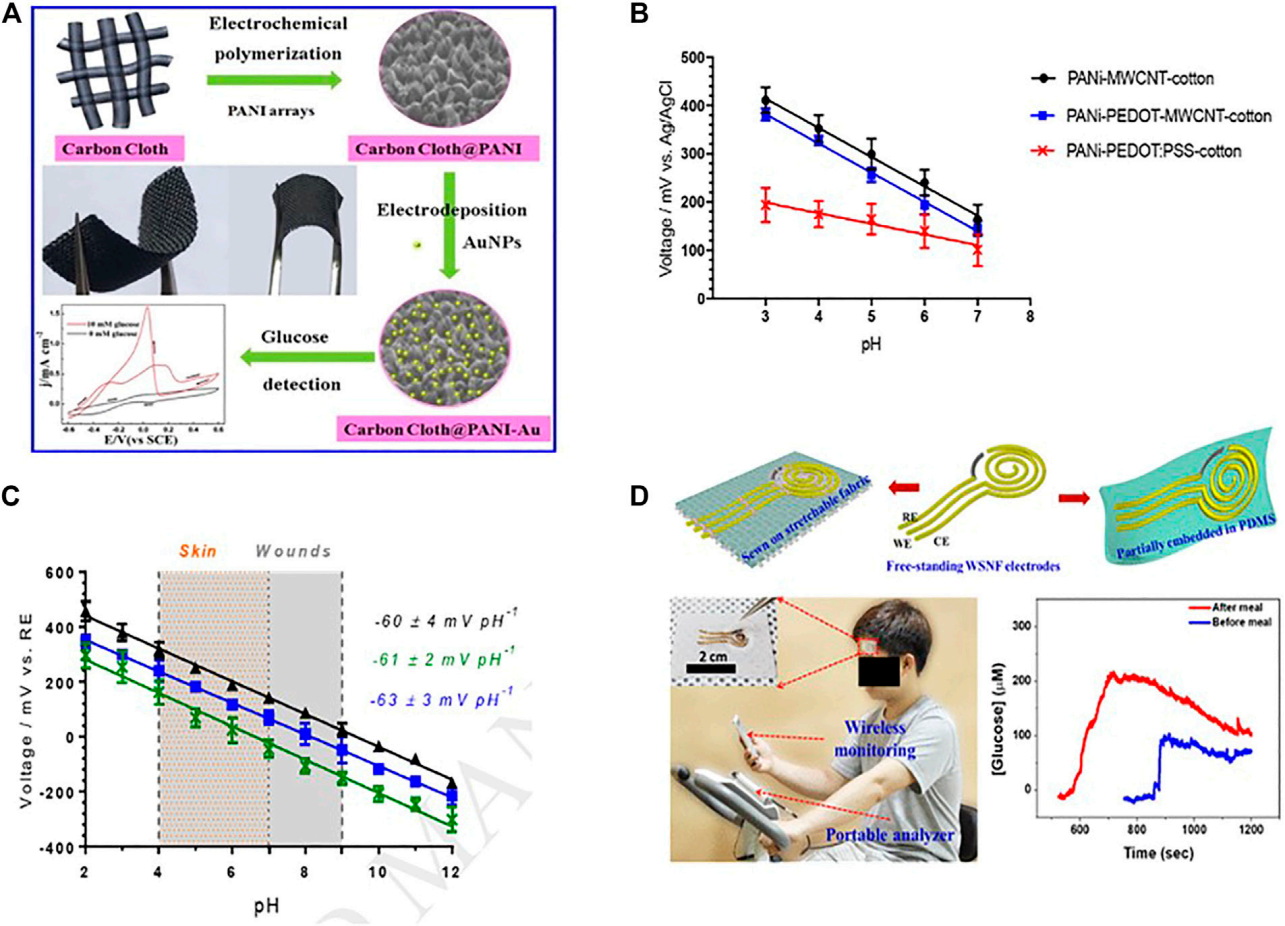
**(A)** AuNPs/PANI/CC based non-enzymatic glucose sensor (Copyright 2017; Sensors and Actuators). **(B)** PANi-coated conductive cotton yarns' pH sensitivity, the error bars show the standard deviation of measurement on *n* = 3 replication fibers (Copyright 2019; Sensors and Actuators). **(C)** An open circuit PH study potentiometric response of PANi-PEDOT-MWCNT-cotton electrodes. (Copyright 2019; Sensors and Actuators). **(D)** Schematic illustration of rGO/PU-Au nanocomposite fiber used in manufacturing sweat-based wearable electrochemical glucose (Copyright 2019; American Chemical Society).

Conductive polymer compound with metallic nanomaterials shows enhanced selectivity and stability for measuring metabolites like glucose and lactate. Xu et al. developed a non-enzymatic glucose sensor by using gold nanoparticles, polyaniline arrays, and a carbon cloth electrode (Xu et al., 2017). PANI was first grown vertically on a flexible carbon cloth (CC) electrode surface to form PANI arrays with a 200 nm height and a 100 nm diameter (Figure 6A). And the integrated electrode (AuNPs/PANI/CC) can electrochemically catalyze the oxidation of glucose. The linear range of the flexible non-enzymatic glucose sensor is 10.26 μM to 10.0 mM, with a sensitivity of 150 μA cm^−2^ mM ^−1^ with a detection limit of 3.08 μM (S/N = 3) (Xu et al., 2017).

#### Conductive Polymer Combines With Carbon Nanomaterials

Conductive polymer nanocomposites combine with carbon nano-species such as carbon nanotubes, graphene, and carbon nanofibers have been evolved. These carbon compounds enhance the structural configuration of conductive polymer chains and allow charge carrier passage, which as a result, increases the conductivity. CNT with conductive polymer shows improved sensing properties in electrochemical biosensors with high stability and good selectivity (Rahimi et al., 2017;Zhou et al., 2017). The most Common CNT-based polymer nanocomposite are PEDOT-CNT-CPE, PEDOT-MWCNT, PPy-MWCNT-ITO (Shrivastava et al., 2016). Xu et al. fabricated a nitrobenzene electrochemical biosensor based on a carbon paste electrode, modified with a PEDOT-CNT nanocomposite. This electrode was used to analyze hydroquinone, dopamine, and nitrobenzene (Xu G. et al., 2014). Similarly, K. Sing et al. developed a multi-walled carbon nanotube (MWCNT), polypyrrole (PPY)- *p*-toluene sulfonic acid (PTS) based electrochemical biosensor for cholesterol detection. At 9s, the sensor demonstrates high sensitivity and rapid response (Singh et al., 2012). Recently, Smith et al. created a wearable pH sensor cotton yarn by dipping and drying it in PEDOT: PSS and multi-walled carbon nanotubes (MWCNT), followed by PANI deposition. The graph shows the standard deviation on three different replicant fibres (Figure 6B). This resulted in electrodes with substantial biocompatibility and antibacterial properties, Which could be used in the future to create wearable solid-state pH sensors (along with quasi-reference electrodes) and for real-time wound and skin pH measurement over a broad pH range (2.0–12.0) and achieve a rapid, selective, and Nernstian response (−61 2 mV pH^−1^) (Figure 6C) (Smith et al., 2019).

Polymers combined with graphene (nanocomposites) demonstrate great potential in wearable electrochemical biosensors. This composite modified electrode has combined graphene’s excellent conductivity and the advantages of polymer nanomaterials, which can increase the durability, biocompatibility and sensitivity of sensors (Xu G. et al., 2014; Hou et al., 2017; Toi et al., 2019). A highly sensitive fiber optic pH sensor based on reduced graphene oxide-polyaniline (rGO-Pani) nanocomposite is fabricated and characterized using the SPR technique. The *in-situ* approach was used to successfully synthesize the rGO-Pani nanocomposite. The sensor’s output is outstanding at low and high pH levels, with a maximum sensitivity of 75.09 nm/pH at pH 11.35 (Semwal and Gupta, 2019).

Phan et al. demonstrated a nonenzymatic wearable patch for on body glucose sensing based on polyurethane (PU) and reduced graphene (rGO) composite fiber, which was further modified with oxygen-containing functional groups. The wearable glucose sensor is highly sensitive (140 mA L mmol^−1^cm^−2^), with a low detection limit of 500 nmolL^−1^. Furthermore, due to the high rGO-PU fabric stretchability, the proposed wearable glucose sensor could be stretched up to 30% and had a high mechanical resilience under repeated cycles of deformation (Figure 6D) (Toi et al., 2019).

#### MXene Based Electrochemical Biosensors

In addition to the above-mentioned sensing nanomaterials, a new class of 2-D material known as MXene has recently arisen as an inorganic compound, consisting of nitrides, transition metal carbides, or carbonitrides (Ti_3_C_2_T_x_) (Guo et al., 2019). It has a thickness of several atomic layers, excellent conductivity, a wide surface area, and excellent biocompatibility, which endows a great prospect in the field of electrochemical biosensors (Yoon et al., 2020). (Tan et al., 2017; Nayak et al., 2018; Wu et al., 2018; He et al., 2020).

**FIGURE 7:**
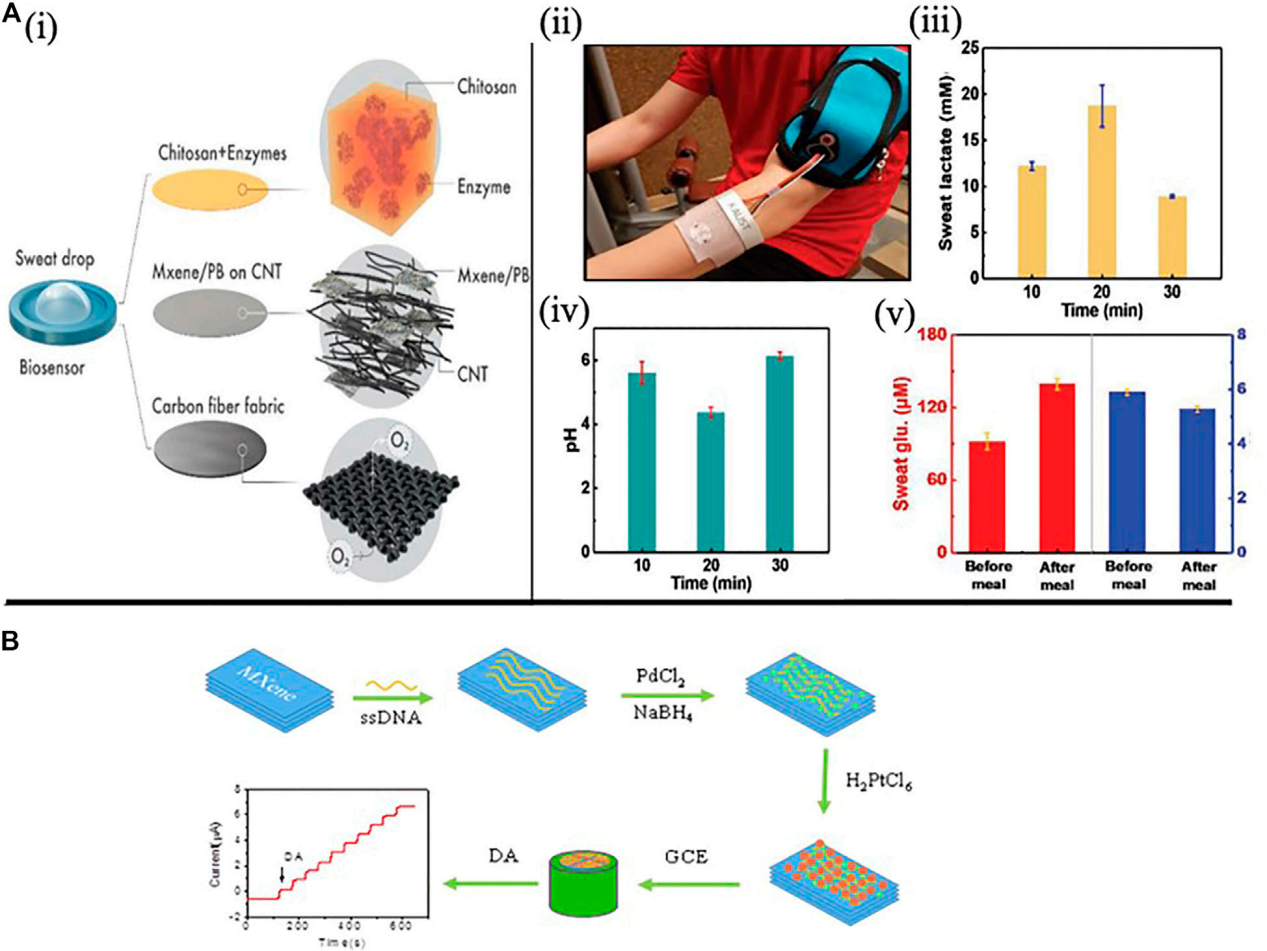
**(A)** a wearable MXene based electrochemical biosensor. i) the oxygen-rich enzyme electrode is depicted schematically. ii) On the skin, sweat-monitoring wearable patch. iii) Comparison of lactate levels at various points during exercise using three separate lactate sensors. iv) Comparison of pH levels at various points in the exercise using three different pH sensors. v) Glucose levels before and after meals were compared using three different glucose and pH sensors. (Copyright 2019; John Wiley and Sons). **(B)** The Ti_3_C_2_/DNA/Pd/Pt nanocomposite fabrication process. (Copyright 2018; Journal of Electroanalytical Chemistry).

The hydrophilic nature of MXene can selectively absorb biomolecules. For instance, Lei et al. proposed a portable multifunctional sweat-based biosensor with 2D MXene for the long-term and subtle identification of biomarkers (such as pH, glucose and lactate) in sweat (Figure 7A). Using artificial sweat, average pH level and electrochemical sensitivity values for lactate 11.4 μA mm^−1^ cm^−2^ and glucose 35.3 μA mm^−1^ cm^−2^ were recorded in three different graphs (Lei et al., 2019). Zheng created sensitive dopamine (DA) sensor using a separate nanomaterial (MXenes/DNA/Pd/Pt), with MXene nanoparticles serving as a conductive matrix for Pd/Pt nanoparticles. The hydrophobic aromatic group adsorbed on the surface of MXenes induces the *in-situ* growth of PdNPs and Pd/Pt nanoparticles (Figure 7B). The sensor shows excellent linearity in the DA concentration range of 0.2–1,000 μM, as well as high selectivity against ascorbic acid, glucose and uric acid (Zheng et al., 2018). MXenes nanosheets also have the ability to strip heavy metals as well as to detect heavy metal ions (e.g., Cu, Li, Na, K atoms) (Guo et al., 2015; Shahzad et al., 2017). Aside from small molecules, metal ions and MXenes combine to have a similar doping effect. Zhu et al. studied the electrochemical reaction of MXene to recognise the coexistence of Cu^2+,^ Pb^2+,^ Hg^2+,^ and Cd^2+^ and suggested a new platform for the detection of high sensitivity metal ions. (Zhu et al., 2017).

## Nanomaterial Applications in Electrochemical Immunosensors and DNA Biosensors

### Electrochemical Immunosensors

**FIGURE 8:**
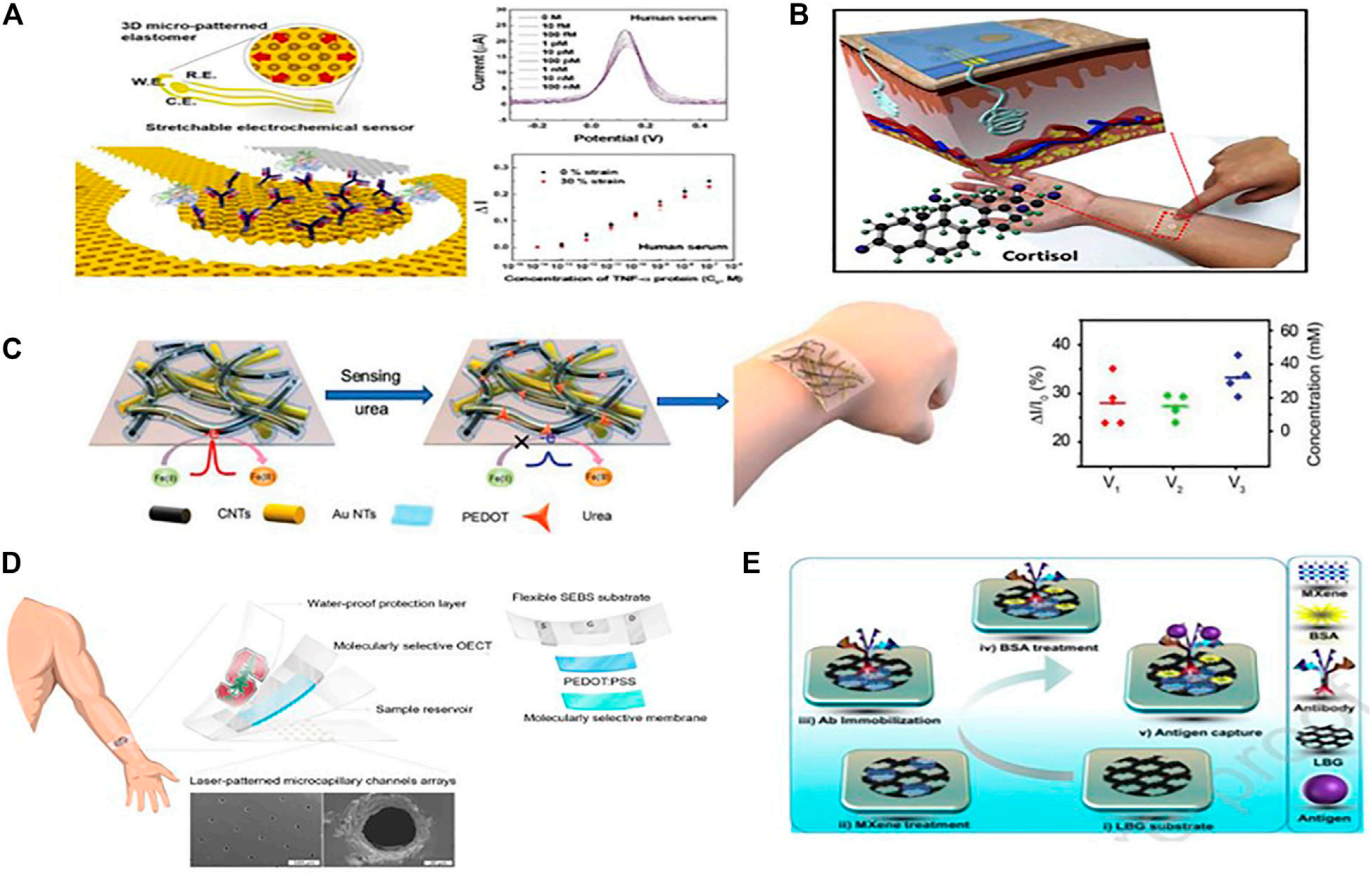
Schematic diagrams of electrochemical immunosensors. **(A)** 3-D micro-patterned elastomeric substrate-based Stretchable electrochemical immunosensor. (Copyright 2019; Sensors and Actuators). **(B)** LOP with microfluidic and electrochemical sensing components for wearable POCT, as well as a schematic diagram of the LOP platform for wearable biomarker detection, fabricated on a mogul-patterned substrate with 3-D nanostructured Au as a working electrode (Copyright 2020; Biosensors and Bioelectronics). **(C)** the development of a flexible sensor for the electrochemical detection of urea (Copyright 2018; American Chemical Society). **(D)** A patch-type wearable cortisol sensor based on carbon nanotubes and a lightweight SEBS elastomer substrate with a PEDOT:PSS semiconductor layer and an MSM. (Copyright 2018; Science Progresses.). **(E)** a Ti_3_C_2_T_x_ MXene-loaded laser-induced graphene cortisol immunosensor (Copyright 2021; Sensors and Actuators).

Electrochemical immunosensors have been extensively used in medical diagnostic and therapeutic applications, doping or impurities, and the food industry to track biological components and biological molecules of environmental origin and influence. where antibodies are probes that form ion complexes with the same antigen pairs with specific targets. Nanomaterials are the best candidates for developing electrochemical immunosensors with good sensitivity and signal transduction capability. A significant number of electrochemical label-free immune strategies have recently been developed and used to identify multiple target biomarkers associated with many kinds of diseases (Tuteja et al., 2018), including viruses (Kaushik et al., 2018), cardiac markers, and other proteins (Dutta and Lillehoj, 2018; Haji-Hashemi et al., 2019). Focused on this label-free method, a non-faradaic impedimetric portable/wearable cortisol sensor was designed using semiconductive MoS2 nanosheets and vertically aligned metal electrodes to calculate cortisol concentration in artificial sweat samples (Kinnamon et al., 2017). As a new immunosensing platform, Stretchable and disposable electrochemical biosensors are eager to identify biomarkers from body fluids such as saliva, interstitial fluids, sweat, or wound fluids, as well as various biomechanical organisms (Kim et al., 2011; Cao et al., 2014; Gao et al., 2016c; Liu et al., 2016; Liu et al., 2017). However, the realization of such stretchable electrochemical biosensors has been narrowed by the challenges of obtaining electrochemical electrodes with high sensitivity, stretchability, and stability during deformation. With that in mind, Bo-Yeong et al. proposed a simple, durable and stretchable disposable point of care electrochemical immunosensor based on metallic nanomaterials. A three-dimensional, micro-patterned stretchable substrate was used to form thermally evaporated gold as working and counter electrodes (Figure 8A). The sensor shows high sensitivity and stability when stretching up to 30%. The sensor can detect low concentrations of target molecules, down to 100 fM of TNF- α protein, an inflammation biomarker**.** After all, it was still challenging to develop a soft, skin-interfaced biosensor patch entirely operative lab-on-patch technology, with particular significance for non-invasive detection and differentiation of biomarkers from body fluids (Heikenfeld et al., 2018, 80; Zhao et al., 2019; Lee et al., 2020). It will also be more user-friendly than lab-based immunoassays or in-hospital (POCT) point-of-care testing, which involves numerous *in vitro* sample processing steps and user expertise in sample analysis (Daniels and Pourmand, 2007). Many current immunoassay procedures use labelling to produce a detection signal, in which an antibody, fluorescent markers, or redox pair is added to the detection probe. These methods are time-consuming, need several steps, and cause discomfort to the wearer (Joung et al., 2019). To meet these challenges, Lee et al. developed a stretchable, wearable lab-on-a-patch (LOP) device made up of a label-free impedimetric biosensor and a stretchable microfluidic system for on-body measurement of the hormone, biomarker, and cortisol. A three-dimensional nanostructured gold was used as a sensing electrode to achieve the high sensitivity required to measure the pM-levels of cortisol in sweat (Figure 8B). Using an antibody as a probe biomolecule, this biosensor measured sweating cortisol accurately during exercise, ranging from 1 pg/ml to 1 μg/ml, under a 30% strain (Lee et al., 2020). This LOP platform may be enhanced to track other biomarkers in sweat such as cytokines, neuropeptides, therapeutic drugs in sweat and a broader range of biomarkers in other biofluids such as interstitial fluids or wound exudate. Further, we see a great roll of nanomaterials in molecular imprinted polymer-based sensors (MIP’s). Huang et al. recently proposed a flexible electrochemical urea sensor (Figure 8C). In their first work, they developed the MIP by imprinting urea with electropolymerized (PEDOT) on a network of carbon nanotubes and gold nanotubes (AuNTs) (Liu et al., 2018). The developed flexible sensor demonstrated a strong linear response to physiologically significant urea levels while showing negligible cross reactivity. Their second work fabricated an ECL sensor by coating a specific MIP layer on highly luminescent nanospheres immobilized AuNTs networks (Parlak et al., 2018). The sensor can detect lactate and urea from sweat accurately with high stability. Parlak and his colleagues created a wearable organic electrochemical system focused on a molecularly selective nanoporous membrane for non-invasive cortisol sensing (Figure 8D) (Parlak et al., 2018). The cortisol biorecognition is based on a MIP membrane with a laser-patterned microcapillary channel array for sample acquisition and organic electrochemical transistors (OECTs) based on PEDOT:PSS. The wearable sensor’s stability and stretchability are offered by the styrene-ethylene-butylene-styrene (SEBS) elastomer substrate. Lately, laser-burned graphene (LBG) has been developed as an excellent electrode matrix for wearable electrochemical sensing applications because of its one-shot fabrication and excellent electrochemical performance (Ugur et al., 2014;Hamblin, 2016). Jong et al. recently proposed a Ti_3_C_2_T_x_ (MXene/LBG) based wearable electrochemical impedimetric immunosensor with a 3-D electrode network for noninvasive cortisol biomarker identification in human sweat at the point of treatment (POC). Laser-induced graphene (LBG) is the basic material used in electrode construction since it is stable and has strong electrical properties. Ti_3_C_2_T_x_ MXene, which has excellent electrochemical properties and outstanding enzyme loading capabilities, was deposited on the electrode (Figure 8E). The cortisol sensor had a very low concentration limit of 3.88 pM and excellent selectivity. This MXene LBG-based flexible noninvasive patch can be used to identify other biomarkers or pathogens. The developed path can be coupled with a wearable electrochemical front-end for impedance signal monitoring and wireless data transmission for smartphone-based biomarkers or pathogen diagnosis properties (San Nah et al., 2021).

### DNA Biosensors

Conductive nanomaterials and nanocomposites have lately gained popularity as potential materials for DNA biosensors. Electrochemical biosensors for DNA detection are being developed and used in many human applications such as food, healthcare, environment, etc. (Ramanavičius et al., 2006; Booth et al., 2011). A DNA biosensor is designed by immobilizing a DNA probe on an electrode surface and then using hybridization to label the target DNA sequence. This hybridization, known as transduction in the technological era, can be observed optically and electrochemically. A DNA biosensor for detecting the H5N1 gene sequence of the influenza virus was created using a DNA aptamer immobilized hybrid nanomaterial-modified (MWCNT/PPy) electrode. The modified electrode nanoparticles provide a porous structure with a wide effective surface area. The latest (PANI-AuNPs) nanocomposite based DNA biosensor detected micro RNA-16 using a streptavidin-AP conjugate to biotinylated recognition sites via transduction with a 0.1 nM detection limit (Saberi et al., 2013). Very recently, for the first time, Jiang and his team proposed a cloth-based DNA biosensor by using nanocomposite (CdTe-MWCNTs) to get a stronger electrochemical signal. Under ideal conditions, target DNA samples (75-bp DNA fragments produced by PCR amplification) were determined in a range of 20 fM to 5 nM, with a detection limit of 8.74 fM and relative standard deviations of 2.04 and 4.75 percent for intra- and inter-assays at 50 pM TD, respectively (Jiang et al., 2020).

## Present Challenges and Future Prospects

Wearable electrochemical biosensors are essential for continuous health and fitness tracking and some other sports applications. The conductive nanomaterials, which are the key components, determine the performance and practical use of such sensors considerably. However, in recent years, emerging conductive nanomaterials have been studied and used to develop wearable electrochemical biosensors due to characteristics like large specific surface area, high porosity, high sensitivity, and selectivity. In this study, different kinds of conductive nanomaterials for sensing electrodes of wearable electrochemical biosensors are summarized.

## Present Challenges

Over all, carbon-based nanomaterials (CNTs, graphene, etc.) have been widely used in the fabrication of sensing electrodes for wearable electrochemical biosensors because of their advantages of good electrical conductivity, high biocompatibility, and low cost. However, for metabolite (glucose) detection, glucose oxides (GOD) normally need to be modified on the working electrode as the carbon-based nanomaterials can not catalyze glucose directly. Consequently, the decrease in the life span of the wearable electrochemical biosensor for detecting glucose caused by enzyme inactivation is the main issue limiting its broad applications and commercialization. Metal-based nanomaterials, particularly noble-based metals, having great and promising electrocatalytic properties, particularly in wearable glucose non-enzymatic sensors. They can detect glucose directly without GOD, demonstrating good stability. But the main issue is cost, which is high. Cu nanowires have been studied as a potentially promising material because of their low cost and high conductivity. Still, the weak stability against oxidation and chemical corrosion, as well as the final decrease in conductivity over time, may limit its practical applications for wearable electrochemical biosensors. The use of conductive polymer nanoparticles in wearable electrochemical biosensors aims to improve sensor flexibility in order to ensure optimal sensor performance after mounting on the human body. The output properties of wearable electrochemical biosensors will not vary because of diverse human body motions. As a result, the catalytic property was not as good as that of metal-based nanomaterials.

### Future Prospects

Wearable electrochemical biosensors can be manufactured from a variety of nanomaterials depending on the needs of real-time applications. Nowadays, more conductive nanomaterials will be developed and used to generate new sensing electrodes for wearable electrochemical biosensors. The new development trends of sensing nanomaterials in the future will be as follows.’

Firstly, sensing nanomaterials with porous structures, high electrical conductivity, and catalytic activity will be often used in both enzymatic and non-enzymatic sensors to enhance the sensor sensitivity. Second, sensing nanomaterials should have superior mechanical properties in order to improve the sensor’s durability and flexibility throughout everyday activities. Third, because there are different interferences (such as glucose, lactate, Na+, K+) in body fluids, nanomaterials with specific identification of analytes like glucose or lactate may attract more attention to increase the selection of sensors. Finally, to produce low-cost, high-performance, and reliable wearable electrochemical biosensors and biosensors, more knowledge of nanomaterial characteristics, as well as advancements in manufacturing and processing procedures, is required. For example, replacing Ag with Cu or carbon-based components and using low-cost CNT synthesis and processing methods. Only preliminary studies on the biocompatibility of nanomaterials for wearable applications have been conducted. More methodical reports on nanomaterials' long-term biocompatibility are urgently needed to support the practical applications of nanomaterials. Graphene has been used to create a variety of wearable electrochemical biosensors, especially for healthcare applications. To ensure high efficiency, additional efforts should be made to improve large-scale advancement with reasonable uniformity and defect-free deposition onto different substrates with high-pitched consistency and yield. However, nanotechnology applications are beginning to emerge, and more research is needed to obtain novel results and uses.
